# Whitefly endosymbionts: IPM opportunity or tilting at windmills?

**DOI:** 10.1007/s10340-021-01451-7

**Published:** 2021-11-02

**Authors:** Milan Milenovic, Murad Ghanim, Lucien Hoffmann, Carmelo Rapisarda

**Affiliations:** 1 Environmental Research and Innovation Department (ERIN), Luxembourg Institute of Science and Technology (LIST), 41, Rue du Brill, L-4422 Belvaux, Luxembourg; 2 Dipartimento di Agricoltura, Università degli Studi di Catania, Alimentazione e Ambiente (Di3A), via Santa Sofia 100, 95123 Catania, Italy; 3 Department of Entomology, Volcani Center, ARO, HaMaccabim Road 68, PO Box 15159, 7528809 Rishon Le Tsiyon, Israel

**Keywords:** Endosymbionts, Symbionts, Bacteria, Whitefly, IPM, *Bemisia*

## Abstract

Whiteflies are sap-sucking insects responsible for high economic losses. They colonize hundreds of plant species and cause direct feeding damage and indirect damage through transmission of devastating viruses. Modern agriculture has seen a history of invasive whitefly species and populations that expand to novel regions, bringing along fierce viruses. Control efforts are hindered by fast virus transmission, insecticide-resistant populations, and a wide host range which permits large natural reservoirs for whiteflies. Augmentative biocontrol by parasitoids while effective in suppressing high population densities in greenhouses falls short when it comes to preventing virus transmission and is ineffective in the open field. A potential source of much needed novel control strategies lays within a diverse community of whitefly endosymbionts. The idea to exploit endosymbionts for whitefly control is as old as identification of these bacteria, yet it still has not come to fruition. We review where our knowledge stands on the aspects of whitefly endosymbiont evolution, biology, metabolism, multitrophic interactions, and population dynamics. We show how these insights are bringing us closer to the goal of better integrated pest management strategies. Combining most up to date understanding of whitefly–endosymbiont interactions and recent technological advances, we discuss possibilities of disrupting and manipulating whitefly endosymbionts, as well as using them for pest control.

## Key message

- Whiteflies host the largest number of recorded endosymbiotic bacteria in a single insect
- Endosymbionts enable whitefly’s survival as a phloem feeder and are modifiers of its biology
- Tight integration with the whitefly makes them a very appealing target in whitefly control
- Despite the intensive research, their use in whitefly IPM strategies is still an elusive goal
- We present and discuss the current state of the art of whitefly endosymbionts research

## Introduction

Whiteflies are one of the most economically important groups of pests with global distribution and very wide range of host plants (Kanakala and Ghanim 2019). They cause direct damage by feeding on the plant phloem, and often even more importantly, by vectoring plant viruses of high economic impact. In higher densities, additional damage is caused by sooty mold which grows on honeydew that whiteflies excrete. Whiteflies cause devastating damage both in open-field production and in greenhouses. Global population dynamics of whiteflies is characterized by a history of devastating invasions of ever new regions, which bring along outbreaks of whitefly transmitted viruses in wider areas of the world (de Moraes et al. 2018; Legg et al. 2014). Ongoing climate change modifies the conditions that determine which whitefly species are the fittest for a region. As a consequence, a shift in species composition occurs with certain whitefly species declining and others expanding farther into previously unsuitable regions, giving rise to new invasions (Aregbesola et al. 2019). Control of whiteflies is made challenging by the increasing prevalence of insecticide-resistant populations (Horowitz et al. 2020). Managing whitefly-borne viruses is an even greater challenge, as viruses can be transmitted faster than insecticides are able to act (Lapidot et al. 2014). Biocontrol methods can be effective in reducing whitefly numbers in greenhouses but are not easily applicable in the open-field systems (Gerling et al. 2001). Therefore, more effective, targeted, and sustainable whitefly control methods are highly needed.

A potential exists to develop new whitefly control methods based on their endosymbiotic bacteria. To understand the scope and feasibility of this potential, we need to understand the nature of whitefly–endosymbiont interactions. Feeding exclusively on the phloem sap presents a nutritional challenge that all phloem feeders must overcome. Plant phloem sap is scarce in essential amino acids which phloem feeders need in order to build their proteins (Douglas 2006). Carotenoids, which play important roles for insects for functions such as vision, defense, signaling, and antioxidative protection, are also absent from the phloem feeders’ diet (Heath et al. 2013). In the case of whiteflies, the carotenoid biosynthetic pathway is also absent from their genome (Chen et al. 2019, 2016; Xie et al. 2017). The universal solution to this challenge is endosymbiosis with bacteria that complement the host’s metabolism and compensate for the unbalanced diet. For the *Bemisia tabaci* (Gennadius) species group, this solution involves at least three separate genomes in a single individual and ensures that endosymbionts are intracellular across whitefly generations (Luan et al. 2018; Santos-Garcia et al. 2012; Sloan and Moran 2012). Besides the whitefly genome and bacterial genome of the obligatory endosymbiont, the genome of endosymbiont-harboring cells is distinct from the rest of whitefly cells.

The journey that led to these discoveries started in 1912, when German zoologist Paul Buchner, the founder of systematic symbiosis research, first described that whiteflies possess cells that contain endosymbiotic microorganisms. In the era that predates electron microscopy, confocal microscopy, and DNA sequencing, he was able to observe how entire cells containing whitefly endosymbionts are maternally inherited (Buchner 1918, 1912). This report was well ahead of its time, and the next published study on whitefly endosymbionts would not come until 1992, when Clark et al. performed the first molecular identification of whitefly endosymbionts (Clark et al. 1992). It revealed that the primary endosymbiont of whiteflies belongs to a lineage distinct from other sap-sucking insects and that whiteflies can have additional, secondary, endosymbiotic bacteria. Just three years later, Costa et al. (1995) reported that whiteflies can in fact harbor more than one secondary endosymbiont. The latter authors conclude by expressing their expectation that “this field of research will increase in importance because modification of endosymbionts will enable development of novel ways to control insects,” which, together with technological advances in the fields of molecular biology, launches the international efforts to understand whitefly endosymbionts and their potential exploitation. Twenty-five years later, endosymbiont-based insect control remains an elusive goal, and research on secondary endosymbionts seems to provide more questions than answers despite a significant body of literature.

**Fig. 1:**
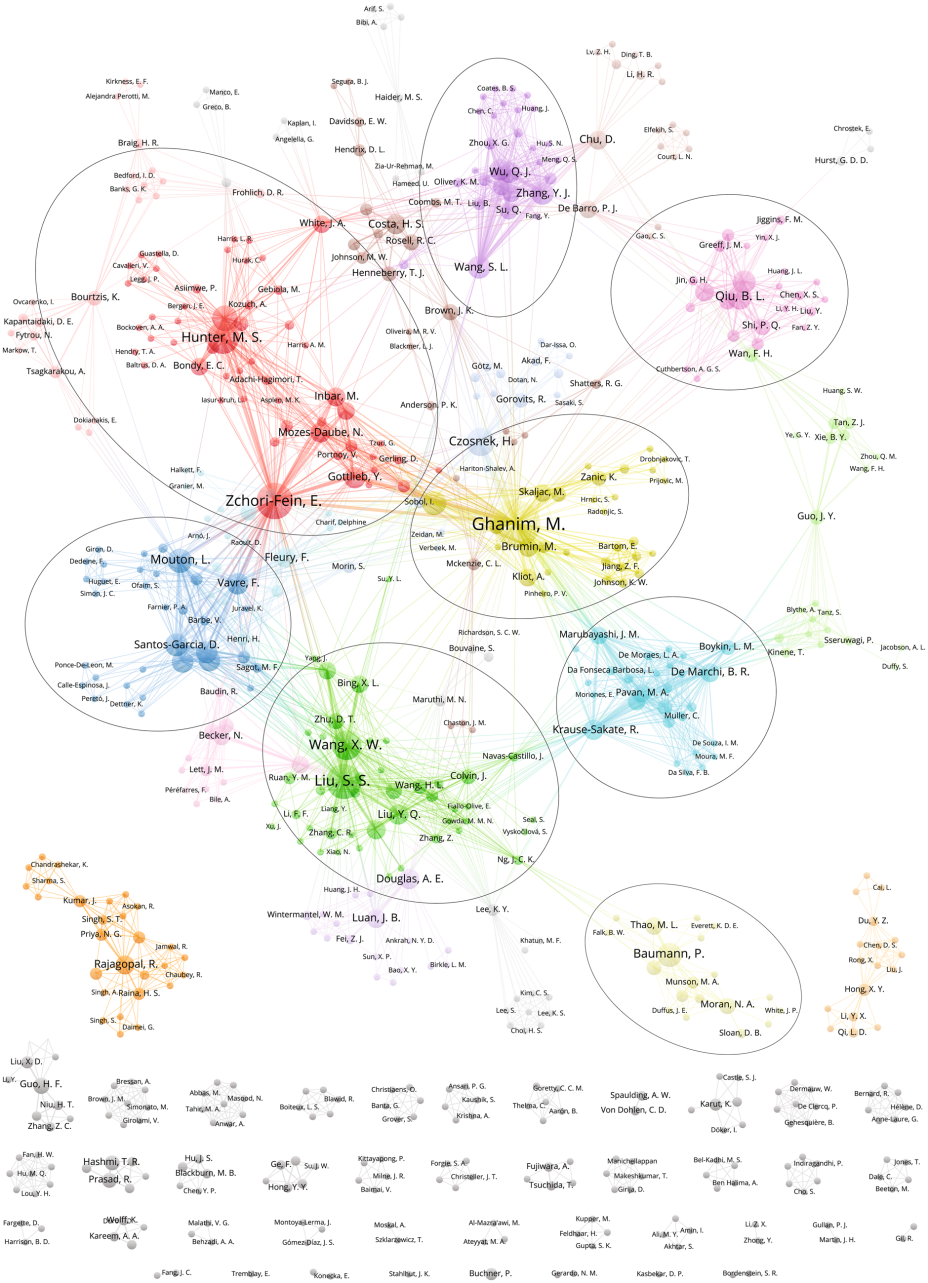
Co-authorship network of whitefly endosymbiont research community. Authors are represented as nodes linked by publication co-authorship. Link strength (line thickness) is proportional to the number of co-authored publications. Eight major clusters (teams) can be identified based solely on co-authorship using VOSviewer clustering and layout algorithm with default settings. Figure was generated using VOSviewer v1.6.14 software

Today, there are over 250 research articles touching on the topic from nearly 700 authors. To help visualize the community of whitefly endosymbiont researchers, in Fig. 1 authors are represented as nodes linked by publication co-authorship using VOSviewer layout and clustering method (van Eck and Waltman 2010; Waltman et al. 2010). Link strength (line thickness) is proportional to the number of co-authored publications. Eight major clusters (teams) can be identified based solely on co-authorship, and not institutional affiliation. This visualization presents an overview of the key researchers and teams in the area of whitefly endosymbionts, which can be very valuable for the readers outside the whitefly research community, as well as to those who are just entering this research field. The whitefly endosymbiont research network is a collaborative one with only two minor disconnected clusters. However, the absence of direct links between some major clusters shows the potential for new collaborations and further acceleration of the research.

This review aims to introduce the reader to the most diverse endosymbiont community explored to date and its implications on the biology of its hosts. Although biology, evolution, and plant virus interactions of whitefly endosymbionts have recently been reviewed by Andreason et al. (2020), the literature on metabolic potential of whitefly endosymbiotic bacteria, and molecular interactions in the endosymbiont-whitefly-plant-virus system is still scattered across experimental articles. In the same time, good understanding of endosymbiont roles and processes that they take part into is essential for their exploitation in the control of whiteflies and whitefly-borne viruses. We review the scientific output of the above illustrated research network to present the current state of the art for each endosymbiont in terms of their evolution, biology, metabolism, and significance for future whitefly control strategies in the framework of IPM in agricultural production.

## *Candidatus* Portiera aleyrodidarum: whitefly’s approach to phloem feeding

Whiteflies are able to source both missing essential amino acids and carotenoids from their close relationship with *Candidatus* Portiera aleyrodidarum (Luan et al. 2015; Santos-Garcia et al. 2015, 2012; Sloan and Moran 2012; Xie et al. 2012).

Portiera aleyrodidarum belongs to the γ-Proteobacteria class of phylum Proteobacteria and is currently the only species in its genus, solely, and always, detected in whiteflies. Its closest related bacteria *Candidatus* Carsonella ruddi and *Candidatus* Evansia muelleri are primary endosymbionts of psyllids and moss bugs, respectively (Kuechler et al. 2013). The closest free-living relative identified to date is *Zymobacter palmae* Okamoto et al. (1993), first isolated from palm sap in Japan, which might have evolved from the same common ancestor (Thao and Baumann 2004a; Okamoto et al. 1993). If the common ancestor was also endophytic, it would explain its acquisition by an ancestral sap-sucking insect. This ancestor would not have been a whitefly, as phylogenetic studies tell us that this association began in ancestral Psyllinea, before the split of whiteflies and psyllids, about 200 million years ago (Santos-Garcia et al. 2015). The two symbiotic organisms have evolved and speciated together ever since. Using sequences of eight Portiera genes, Santos-Garcia et al. (2015) estimated divergence of *B. tabaci* MEAM1 and MED to 380,000 to 100,000 years ago, depending on the phylogenetic test, which is around their *B. tabaci* mtCOI-based 210,000 years ago estimate. In a more recent study (Santos-Garcia et al. 2020), Portiera sequences have been used to resolve long standing inconsistencies in the taxonomy of whiteflies. By using Portiera genomes across 25 whitefly genera, it was possible to infer relationships which have proven difficult to resolve by whitefly sequences alone.

As a result of the very close and long-lasting relationship, Portiera is dependent on the metabolism of its host and has not been cultured to date (Baumann 2005). At the same time, whiteflies cannot survive without Portiera as it was shown in several antibiotic elimination studies (Zhao et al. 2019; Shan et al. 2016; Zhang et al. 2015). By continuous feeding on an antibiotic containing diet, Portiera-free adults were obtained and oviposited a small number of endosymbiont-free eggs which subsequently failed to hatch (Zhang et al. 2015). Failure to hatch likely resulted from the inability of Portiera-free adults to produce viable eggs, which is unsurprising considering the essential nutritional benefits Portiera provides to whiteflies. While different antibiotics and application methods have varying success in eliminating endosymbionts, studies show that Portiera is the least sensitive and most difficult to eliminate (Zhao et al. 2019; Lv et al. 2018; Shan et al. 2016; Raina et al. 2015; Zhang et al. 2015; Xue et al. 2012; Ahmed et al. 2010b; Costa et al. 1997). Antibiotic treatment practically demonstrates that elimination of Portiera indeed leads to the elimination of whiteflies. While being a useful demonstration of potential benefits of eliminating Portiera, use of antibiotics is unfortunately unsuitable as a direct control method for the same reasons that use of antibiotics in plant production overall is controversial (Vidaver 2002). Whitefly control based on endosymbiont disruption has not been demonstrated in the field conditions to date. That is not to say that there are no successful field experiments in other insects. It has been shown in the field trials that disruption of *Candidatus* Erwinia dacicola, endosymbiont of olive fly, *Bactrocera oleae* Rossi, using copper compounds is possible and that population of the pest was consequentially reduced (Bigiotti et al. 2019).

It was long considered that Portiera is a Gram-negative bacterium with only one cell membrane, which presented an apparent contrast to many other obligatory endosymbionts, including its closest relatives *Ca.* Evansia muelleri and *Ca.* Carsonella ruddi (Santos-Garcia et al. 2014b; Baumann 2005). This aspect was revisited by Santos-Garcia et al. (2014b), who performed transmission electron microscopy studies and identified a typical three membrane system commonly seen in other endosymbionts (two membranes synthetized by Portiera and one from the host cell vesicle). Difficulties in detecting the typical double membrane result from a very limited amount, or perhaps absence, of peptidoglycan in the periplasmic space, which makes the space very fragile and challenging to observe. This is in accordance with the lack of a peptidoglycan synthetic pathway in the Portiera genome (Santos-Garcia et al. 2014b).

**Fig. 2:**
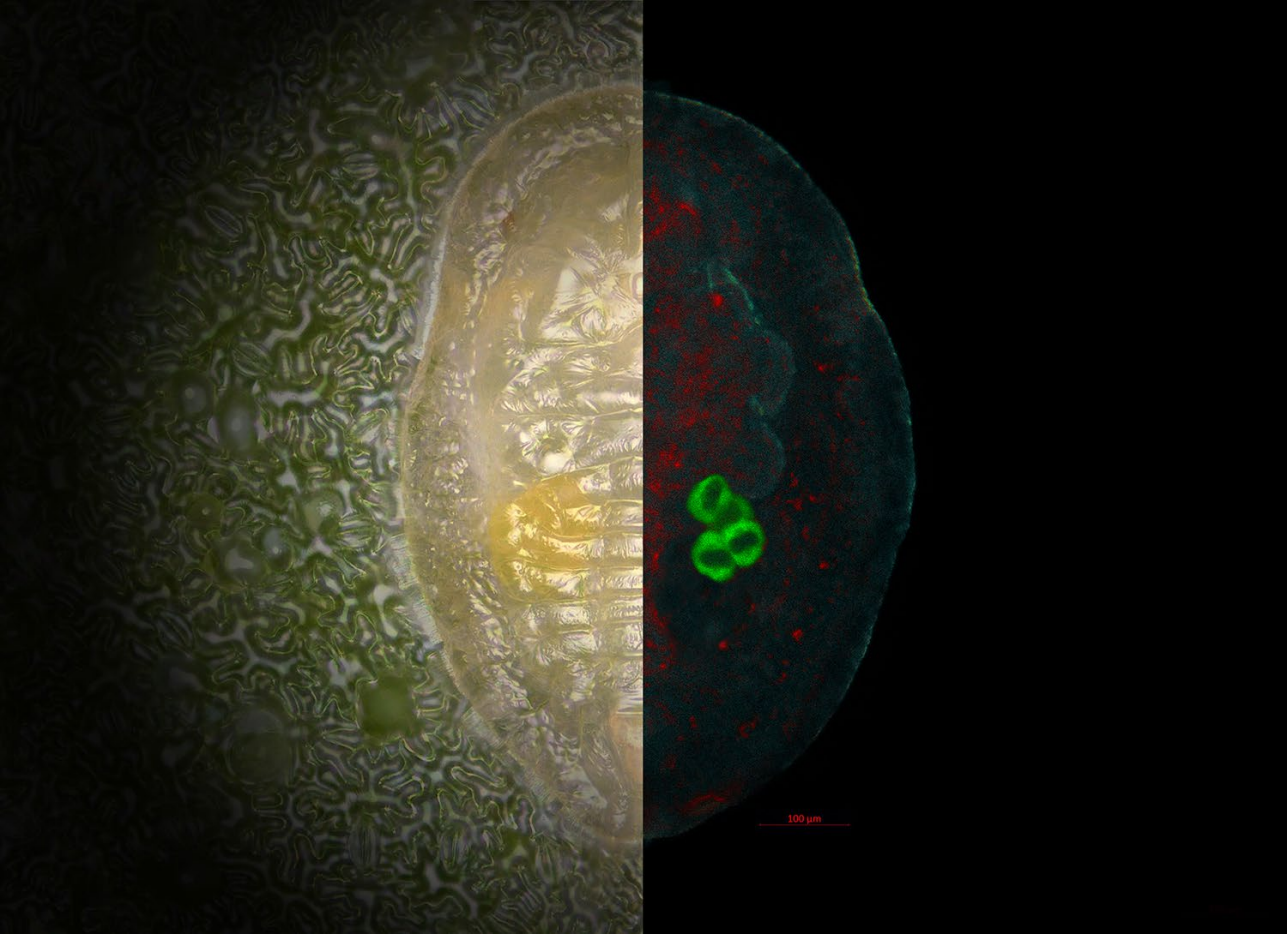
Nymph of *Bemisia tabaci* MED on tomato leaf imaged under optical microscope (left) and laser scanning confocal fluorescence microscope (right). In fluorescence microscope image, green color represents fluorescence of Cy3 labeled Portiera-specific DNA probe, while red represents Cy5 labeled Rickettsia-specific DNA probe. Light blue represents cuticle autofluorescence signal when excited with 405 nm laser line. Fluorescence in situ hybridization was performed according to protocol described by Brumin et al. (2012). In optical microscope image, location of bacteriocytes is easily recognized through the translucent cuticle due to their high carotenoid content which results in yellow appearance. In fluorescence image, four bacteriocyte cells can be seen clearly due to the high density of labeled Portiera. Image composite was generated using Zeiss ZEN Black 3.3 and Adobe Photoshop CC

Whiteflies have taken extra precautions to protect the bacteria they depend on. This bacterial symbiont is located inside of specialized host cells called bacteriocytes where they are contained within vesicles made from the host cell membrane (Szklarzewicz and Moskal 2001; Costa et al. 1993). Bacteriocyte cells form two symmetrical organs called the bacteriome, formerly known as mycetomes, which are easily observed during nymphal stages due to their high content of carotenoid pigments (Fig. 2). Inheritance of Portiera is guaranteed with the process where entire bacteriocyte cells from the mother are transferred to the eggs (Luan et al. 2018; Funkhouser and Bordenstein 2013; Szklarzewicz and Moskal 2001; Buchner 1912). Endosymbionts do not have any extracellular phase during this highly regulated process of their transfer to the oocyte which involves major changes in whitefly gene expression, cell division, cell adhesion, and mobility (Luan et al. 2016; Coombs et al. 2007). In *Aleyrodes proletella* (L.), several bacteriocytes are transferred to the oocyte, which are subsequently degraded during the embryonic development, releasing the endosymbionts only after entering the oocyte, which are then incorporated into newly formed bacteriocyte cells (Buchner 1953). Similar transfer of intact bacteriocytes has also been described in cockroaches and sucking lice (Douglas 1989). On the other hand, in *B*. *tabaci*, precisely one bacteriocyte cell is transferred to the posterior pole of each oocyte which divides during the embryonic development, in contrast to their degradation in *A. proletella* (Luan et al. 2018; Costa et al. 1996; Tremblay 1959). These cells continue to divide without degradation during the development of *B. tabaci* and form two bacteriocyte organs. Persistence of inherited bacteriocytes has to date only been observed in whiteflies, more specifically, in *Bemisia* genus (Luan et al. 2018; Coombs et al. 2007). During the adult development, a portion of bacteriocyte cells is associated with the ovaries to be individually incorporated in each formed oocyte and eventually the fertilized egg. Such maternal inheritance means that bacteriocytes are expected from genetic recombination and have different genomes than other somatic cells. This was demonstrated by microscopy observations, microsatellite marker assays, and separate genome sequencing of bacteriocytes and whitefly heads (which do not contain bacteriocytes) (Luan et al. 2018). Genomes of bacteriocytes and other somatic cells are identical in non-*B. tabaci* whiteflies as a consequence of bacteriocyte degradation in the egg (Xu et al. 2019). The same team of researchers also reported preliminary evidence of polyploidy in bacteriocyte cells in *B. tabaci*. More work is also needed to reconstruct the ancestry of these maternally inherited cells, specialized for hosting and inheritance of whitefly endosymbionts (Luan et al. 2018). The strictness of this vertical transmission and single, ancestral Portiera infection is further validated by comparing phylogenetic trees constructed from whitefly sequences with those constructed from Portiera sequences (Santos-Garcia et al. 2015; Thao and Baumann 2004a; Campbell 1993).

The genome of Portiera has been severely reduced during its co-evolution with the host and is around 350,000 base pairs long and with only around 300 genes (Zhu et al. 2019; Jiang et al. 2012; Santos-Garcia et al. 2012). Its genes code enzymes involved in essential amino acid synthesis, carotenoid synthesis, information transfer, and oxidative phosphorylation (Calle-Espinosa et al. 2016; Santos-Garcia et al. 2012; Sloan and Moran 2012). The reduction of the Portiera genome went even further than the loss of non-essential pathways. This reduction has been described as extreme even for endosymbionts, as it is missing both citrate cycle and glycolysis pathway components (Opatovsky et al. 2018). Its energy metabolism was recently reconstructed and revealed highly unusual adenosine triphosphate (ATP) generation through oxidative phosphorylation coupled to amino acid and carotenoid synthesis (Calle-Espinosa et al. 2016). Even the essential amino acid and enzyme cofactor synthesis pathways are incomplete in Portiera and are complemented by the genes in the whitefly genome (Luan et al. 2015; Upadhyay et al. 2015). At least ten of those homologues were shown to be of bacterial origin, from at least four different bacterial lineages (Luan et al. 2015).

Metabolic modeling of Portiera-*B. tabaci* interactions suggests that Portiera exchanges 49% of the metabolites it produces with its host, with amino acids comprising 40% of the metabolic flux (Ankrah et al. 2017). Three essential amino acids that Portiera over-produces and exports to the host according to the same modeling study are tryptophan, threonine, and methionine. Portiera’s role in lysine production necessitates a deeper look: within whiteflies, the genome of *B. tabaci*-associated Portiera is even more functionally reduced than in other species, with fewer genes, significant genome rearrangements, and lower coding density despite being ~ 70 kb longer (Santos-Garcia et al. 2015; Sloan and Moran 2013). As an example, Portiera isolated from the whiteflies *Aleurodicus dispersus* Russell, *A. floccissimus* Martin et al., and *Trialeurodes vaporariorum* Westwood has a complete set of genes necessary for lysine synthesis. On the other hand, Portiera associated with species of the *B. tabaci* complex is missing three enzymes of this pathway (dapB, dapF, and lysA), which it complements from the horizontally acquired homologues in the host genome (Luan et al. 2015; Rao et al. 2015b). These homologues are not present in currently sequenced whiteflies outside of the *Bemisia tabaci* complex (Luan et al. 2015). The high level of genome instability in Portiera seems to have evolved around 70 million years ago in the common ancestor of Aleurolobini tribe (Santos-Garcia et al. 2020). This point in time is very close to the transition from multiple bacteriocyte inheritance to single bacteriocyte inheritance in whiteflies (Santos-Garcia et al. 2020). Based on the evidence available to this date, it is hard to say which event caused the other, and it remains a chicken and egg paradox. Considering that the *B. tabaci* complex is arguably the most successful colonizer of agroecosystems among all whiteflies, it is possible that the success of these species is linked to the tighter relationship with its endosymbionts via more controlled transmission and preservation of a separate bacteriocyte genome.

Loss of most of the metabolic pathways present in free-living bacteria and incomplete metabolic pathways complemented by its host cell indicate that Portiera is on the evolutionary path to becoming a cell organelle, or perhaps more appropriately, a “symbionelle” (Santos-Garcia et al. 2015). A symbionelle is a proposed term to distinguish organelles that predate multicellularity from endosymbionts of multicellular organisms that later evolved to organelles (Reyes-Prieto et al. 2014). The currently accepted threshold for considering an intracellular symbiont as an organelle is that the majority of functional proteins are encoded in nuclear DNA, with only a small portion being encoded in organellar DNA (Cavalier-Smith and Lee 1985). For the moment, Portiera has not reached this threshold. In the primary endosymbiont of aphids, *Buchnera aphidicola* Munson et al. (1991), protein import from the host cell occurs, bringing the endosymbionts of sap-sucking insects closer to crossing the line between endosymbiont and organelle, with some authors proposing that this is enough to say that the border has already been crossed (Santos-Garcia et al. 2015; Nakabachi et al. 2014). It is not very far-fetched to say that Portiera, being even more reduced than *Buchnera*, might in the future be reclassified as cellular symbionelle.

## Whiteflies harbor facultative endosymbionts in addition to Portiera

Portiera is not the only endosymbiont that whiteflies harbor and not always the only resident of its bacteriocyte cell. To date, the identified secondary endosymbionts of whiteflies belong to seven genera: *Arsenophonus* Gherna et al. (1991), *Cardinium* Zchori-Fein et al. (2004), *Fritschea* Thao et al. (2003), *Hamiltonella* Moran et al. (2005), *Hemipteriphilus* Bing et al. (2013b), *Rickettsia* da Rocha-Lima, and *Wolbachia* Hertig (Kanakala and Ghanim 2019). Whiteflies of the group *B. tabaci* are the species with the highest number of reported secondary endosymbionts to date (Brumin et al. 2019). All secondary endosymbionts are maternally inherited in whiteflies, although their lifestyles show significant variation. Their localization pattern differs from co-inhabiting the bacteriocyte with Portiera to being present through the body (Shi et al. 2018, 2016; Caspi-Fluger et al. 2011; Gottlieb et al. 2008; Costa et al. 1993). Species composition of whitefly secondary endosymbionts is complex, highly variable over time, and is correlated with and influenced by a number of factors (e.g., whitefly genotype, geographic location, etc.) (Bockoven et al. 2020; Zchori-Fein et al. 2014).

The fact that vertical transmission of secondary endosymbionts is not enough to guarantee their survival in whitefly populations points towards a more complex interaction with their host and environment. This raises an important question about the dynamics of secondary endosymbiont communities: Do whiteflies owe their global success to their diverse community of endosymbionts or are these bacteria taking advantage of a very successful host? Further, can secondary endosymbionts be exploited for the development of new control methods?

We may not have all answers yet, but a rapidly growing output of global research efforts is bringing us ever closer. By synthetizing from the currently published research, we present below the current state of human knowledge on the topic.

### *Candidatus* Hamiltonella defensa

The first facultative endosymbiont to be detected in whiteflies was initially referred to as the “S-Symbiont” of whiteflies, followed by the “T-type endosymbiont” and “pea aphid *Bemisia*-like bacterium” in the aphid community (Baumann 2006; Clark et al. 1992). In 2005, the species was formally named *Candidatus* Hamiltonella defensa in the honor of the evolutionary biologist William D. Hamilton (Moran et al. 2005). It is a Gram-negative bacterium belonging to the family *Enterobacteriaceae* of γ-Proteobacteria and has so far been detected as an endosymbiont of sap-sucking insects including whiteflies, aphids, and psyllids (Shan et al. 2019). In whiteflies, it has been detected in *B. tabaci, T. vaporariorum* (Westwood), and *Siphoninus phillyreae* (Haliday) (Skaljac et al. 2013, 2010). Within *B. tabaci*, it has only been detected in Mediterranean (MED), Middle East-Asia Minor 1 (MEAM1), and New World 2 species (Kanakala and Ghanim 2019). Global phylogenetic studies of Hamiltonella in whiteflies reveal only one group, suggesting a single, ancestral acquisition of this endosymbiont (Kanakala and Ghanim 2019; Rollat-Farnier et al. 2015).

Localization of Hamiltonella using both fluorescence in situ hybridization (FISH) and transmission electron microscopy (TEM) techniques revealed that in species of the group *B. tabaci* it is always co-located in the same bacteriocyte as Portiera (Marubayashi et al. 2014; Gottlieb et al. 2008). Bacteriocyte confinement and low genetic diversity are indicative of very tight association with its host and absence of horizontal transmission. In contrast, Blawid et al. (2015) reported apparent localization of Hamiltonella in mouthparts, reproductive organs, and leg muscle in *B. tabaci* MEAM1. More studies are needed to confirm this phenotype with certainty as single detection in combination with visible cuticle autofluorescence and absence of data on which percentage of individuals showed this signal cast a doubt on the validity of this detection. Exceptionally, in *S. phillyreae* this endosymbiont shows a clearly scattered distribution in the body of adults and nymphs (Skaljac et al. 2013). Absence of sequence data from this *S. phillyreae* strain of Hamiltonella makes it impossible to exclude the possibility that scattered phenotype in this case results from a strain of Hamiltonella that is different from Hamiltonella in other whitefly species.

The genome of the whitefly-associated Hamiltonella is 1.72–1.84 mega base pairs long, with around 1,800 protein-coding genes (Rollat-Farnier et al. 2015; Rao et al. 2012). It encodes genes involved in amino acid synthesis pathways, membrane transport, metabolism of cofactors and vitamins, and genes involved in replication, repair, translation, and carbohydrate metabolism (Rao et al. 2012). Having certain biosynthetic pathways does not necessarily imply benefits to its host. Metabolic modeling suggests that Hamiltonella exchanges only 8% of the metabolites it produces with its host, with 93% of that flux being comprised of central carbon metabolism intermediates (Ankrah et al. 2017).

Hamiltonella lacks many genes in essential amino acid biosynthesis pathways. It necessitates metabolites produced not only by *B. tabaci*, but in the case of chorismate, lysine, and phenylalanine pathways, necessary metabolites are produced only by the primary endosymbiont Portiera (Opatovsky et al. 2018; Rao et al. 2015a). This dependence probably explains why it has the same bacteriocyte-confined phenotype as Portiera. Further, Hamiltonella has functional dapB, dapF, and lysA genes which are absent in Portiera and whitefly species outside of the *B. tabaci* group, which raises the possibility that Hamiltonella complements the genome of Portiera to produce lysine in some whitefly species (Luan et al. 2015). However, as previously mentioned, functions of missing lysine genes in Portiera are performed by horizontally acquired homologues in the genome of *B. tabaci* (Luan et al. 2015). These three homologues in the lysine pathway originate from three different lineages of bacteria (Rickettsiales, Enterobacteriales, and Planctomycetes for dapB, dapF, and lysA, respectively), and not from Hamiltonella, which reinforces the conclusion that lysine synthesis capabilities of Hamiltonella are not critical for *B. tabaci* (Luan et al. 2015). Finally, lysine export capabilities of Hamiltonella to its host are unclear and are dependent on aspartate supplied from its host (Ankrah et al. 2017). As for the other whitefly species, lysine synthesis complementation by Hamiltonella cannot be excluded, despite the perfect complementation of missing lysine genes in Portiera, the facultative nature of Hamiltonella tells us that it is not strictly necessary.

Compared with Hamiltonella associated with aphids, whitefly strains also lack genes related to the production of toxins that offer aphids protection from parasitoids, which implies a different role of Hamiltonella in whiteflies (Shan et al. 2019). Based on its metabolism and its facultative nature, it seems logical to expect that Hamiltonella is closer to being a nutritional parasite rather than a symbiont (Ankrah et al. 2017). In spite of that, whitefly-associated Hamiltonella has lost all virulence factors from its genome and has a severely reduced number of genes involved in environmental sensing (Rollat-Farnier et al. 2015). This is to be expected as a bacterium transitions from a parasitic to a symbiotic lifestyle.

Comparative studies between Hamiltonella-infected and Hamiltonella-free whiteflies show us clear benefits of this endosymbiont: Hamiltonella seems to be able to increase whitefly fecundity, survival, and adult body size while decreasing the development time (Su et al. 2013a). In a follow-up study by the same team, Su et al. show that Hamiltonella suppresses jasmonic acid (JA) defense in tomato, resulting in 20% higher adult survival and a twofold fecundity increase (Su et al. 2015). The mechanism was shown to be triggered by a < 3 kDa non-proteinous molecule in the saliva of Hamiltonella-infected whiteflies and to depend on a functional salicylic acid (SA) pathway in tomato (Su et al. 2015). A positive feedback loop seems to exist as Hamiltonella-infected whiteflies increase SA concentration in tomato, and application of exogenous SA to SA-deficient tomato mutant results in increased Hamiltonella densities (Su et al. 2015). Further supporting this hypothesis are the findings that elevated ozone triggered higher levels of SA in the plant and increased Hamiltonella densities in whiteflies (Hong et al. 2017). In artificial feeding assays, Hamiltonella provides fitness benefits on low nitrogen diet. Nevertheless, these results do not directly translate into the conclusion that Hamiltonella is a nutritional symbiont as phloem composition is very different from sucrose and yeast extract diet mixture, in many more ways than nitrogen content (Su et al. 2014a). Antibiotic treatment in this study may have influenced the results as well, as it is known that rifampicin affects primary endosymbiont densities, gut bacterial population, and potentially also the host directly, all of which could affect the fitness evaluation (Andreason et al. 2020; Zhao et al. 2019; Su et al. 2014a).

Two available studies of reproductive manipulation by Hamiltonella are in conflict, varying from no effect to modifying the sex ratio to 20:1 in favor of males (Shan et al. 2019; Su et al. 2013a). The separately observed differing Hamiltonella infection frequency between female (88.9%) and male (12.5%) field collected whiteflies complicates the interpretation even further as it does not agree with either of the above-mentioned studies (Pan et al. 2012). The most probable explanation seems to be that other factors influenced sex ratio, or that Hamiltonella influences sex ratio only under certain conditions. For example, heat stress has been shown to have a negative effect on bacteriocyte function and proliferation, which reduced transmission efficacy of both Portiera and Hamiltonella (Shan et al. 2017).

Hamiltonella produces the chaperone protein GroEL which is shown to interact with tomato yellow leaf curl virus (TYLCV) particles in the insect hemolymph and protects them from proteolysis by the whitefly immune system, resulting in substantially increased transmission efficacy from under 10% to over 80% (Bello et al. 2020; Hong et al. 2017; Su et al. 2013b; Gottlieb et al. 2010; Morin et al. 2000, 1999). GroEL homologues exist in other endosymbionts, but they do not show similar interaction with TYLCV particles (Gottlieb et al. 2010). Finally, it has been experimentally demonstrated that GroEL from Hamiltonella captures TYLCV virus particles (Gotz et al. 2012). It has been suggested that by this interaction Hamiltonella reduces the negative fitness effects that TYLCV has on whiteflies, making the GroEL-TYLCV interaction beneficial for all three organisms (Kliot and Ghanim 2013). The finding that GroEL protein of Hamiltonella is able to capture TYLCV virions has been successfully exploited in creating virus resistant tomato and *Nicotiana benthamiana* Domin plants by expressing GroEL in the plants (Edelbaum et al. 2009; Akad et al. 2007, 2004).

### *Rickettsia* sp.

In 2006, a gram-negative α-Proteobacterium belonging to the *Rickettsia* genus was detected as an endosymbiont in whiteflies (Gottlieb et al. 2006). In the following decade and a half, it has been a central focus of about 40 scientific publications and demonstrated to have a very different biology and dynamics from other endosymbionts in whiteflies. So far, Rickettsia has been detected globally, in seven *B. tabaci* species (MED, MEAM1, Asia II 1, Asia II 3, Asia II 7, China 1, and Sub-Saharan Africa) (Kanakala and Ghanim 2019). Phylogenetically, there are at least four distinct groups of Rickettsia whitefly endosymbionts, which do not match with the evolutionary history of their host (Wang et al. 2020; Kanakala and Ghanim 2019; Ghosh et al. 2015). Most commonly detected Rickettsia groups in whiteflies are closely related to *Rickettsia bellii* Philip et al. (1983) (Zhu et al. 2016; Gueguen et al. 2009). The exception to this is a very recent detection of *Rickettsia* belonging to the Torix group, and it seems to be more recently acquired by whiteflies than the more widely present *R. bellii*-related groups (Wang et al. 2020). Initially, detection of Rickettsia in whiteflies seemed very exciting from the biocontrol point of view, as Rickettsia infection in other insects is linked with mortality of male offspring and parthenogenesis (Hagimori et al. 2006; Lawson et al. 2001). Further research, that is about to be discussed, shows that Rickettsia in whiteflies does not have the same effect and that its role in whiteflies is quite different.

Localization of Rickettsia is the most diverse and dynamic of any other whitefly endosymbiont, with five different phenotypes. Most commonly, it is detected in a scattered random pattern through the hemolymph of adult and nymphal stages. In this phenotype, it is located outside of bacteriocytes and sometimes concentrated around the gut and follicle cells (Brumin et al. 2012; Gottlieb et al. 2006). Soon after the initial detection, a second, strictly bacteriocyte-confined phenotype, was observed and could not be explained by differing Rickettsia strains. Phylogenetic analyses showed that Rickettsia from both phenotypes group together, even when highly variable portions of the genome are interrogated (Li et al. 2017b; Caspi-Fluger et al. 2011; Skaljac et al. 2010; Gottlieb et al. 2008). More recently, three additional phenotypes were detected, showing the localization in (1) wax glands, (2) female colleterial glands, and (3) the bacteriocytes that scattered through the entire abdomen of the adult female (Shi et al. 2018). Factors behind these localization phenotypes are yet to be identified and could range from environmental to genetic. So far, there are no successful experiments showing a shift between two phenotypes in a given whitefly population, although there is evidence that the scattered phenotype is the “default state”. Namely, Li et al. (2017b) successfully achieved plant-mediated horizontal transfer from whiteflies with both the scattered and confined phenotype into Rickettsia-free whiteflies. Regardless of the Rickettsia phenotype in infested whiteflies, the recipient whiteflies always showed a scattered phenotype. This supports the abovementioned phylogenetic studies and points towards a conclusion that the strain of Rickettsia is not the phenotype determining factor, or at the very least, not the only factor. It is possible that Rickettsia is able to infect bacteriocytes only under specific metabolic conditions, and that is why we find a scattered phenotype. However, this theory would not explain why Rickettsia would completely disappear from the hemolymph in the confined phenotype, or how it is able to enter the bacteriocyte cell that is incorporated in the egg. Alternatively, the localization of Rickettsia might be explained by spatial availability of metabolites and enzymes that Rickettsia needs in order to support its reduced metabolism. A low number of Rickettsia cells might always be present in bacteriocytes, below the detection threshold of the FISH technique. Variable gene regulation of its host, influenced by yet unknown factors, may make bacteriocytes more hospitable, if not the only hospitable place in the whitefly’s body, causing the shift from the scattered to the confined phenotype. While the level of detail needed to confirm this theory is still lacking, we do have evidence that the dynamics of Rickettsia differs between phenotypes. Rickettsia exhibits higher post-eclosion density in confined phenotype, which was matched in the scattered phenotype only after 21 days, without being correlated with densities of Portiera and Hamiltonella (Caspi-Fluger et al. 2011).

Rickettsia is transmitted between whiteflies both vertically and horizontally (Caspi-Fluger et al. 2012; Gottlieb et al. 2006). Vertically, it is maternally inherited in the same manner as the primary endosymbiont, with the difference being that usually right after the egg is laid, Rickettsia leaves the bacteriocytes and disperses through the egg (Chiel et al. 2009a; Gottlieb et al. 2006). Horizontal transmission can occur via two mechanisms. It can be plant mediated, in which Rickettsia cells are injected in the plant phloem during whitefly salivation, after which it is able to move along the phloem and infect other whiteflies by ingestion (Li et al. 2017b; Caspi-Fluger et al. 2012). Transmission via parasitoids of whiteflies has also been hypothesized. Larvae of *Eretmocerus mundus* Mercet developing in Rickettsia-infected nymphs result in infected larval and adult parasitoids. Rickettsia reaches the oocytes of *E. mundus*, but is unable to penetrate them, resulting in the absence of vertical transmission in *E. mundus* (Qi et al. 2019; Chiel et al. 2009b). In fact, Rickettsia might have adaptations to enhance this transmission route, as it has been observed that proliferation of Rickettsia is strongly induced with the beginning of the parasitization process (Mahadav et al. 2008). This transmission between trophic levels, from whitefly to its parasitoid, does not occur if Rickettsia is localized and strictly confined in whitefly bacteriocytes (Caspi-Fluger et al. 2011). Finally, the ability of Rickettsia-infested *E. mundus* to infect whitefly nymphs (that might survive parasitation) has not been experimentally demonstrated.

An overview of the metabolic potential and lifestyle of an organism can be obtained by interrogating its genome. Rickettsia has a 1.2 mega base pairs long genome with about 1300 predicted genes in the *R. bellii* group and 1.5 mega base pairs with 1,446 predicted genes in the Torix group (Wang et al. 2020; Zhu et al. 2016). Most of the genome codes for proteins involved in nucleic acid metabolism, energy metabolism, and cellular membrane biogenesis, while missing most of the genes in biosynthetic pathways commonly present in other Rickettsia (Zhu et al. 2016). It relies on enzyme and metabolite complementation from the whitefly host and Portiera to a great degree. For example, it only possesses the enzyme for the final reaction of branched-chain amino acid synthesis (ilvE), but with metabolic complementation from Portiera, it is able to produce leucine, valine, and isoleucine (Opatovsky et al. 2018). Missing glycolytic pathways show that it relies on its host to produce ATP and is also dependent on NAD + supply from its host (Opatovsky et al. 2018). From a nutritional point of view, Rickettsia is costly to its host, and if it provides any benefits, they are unlikely to be nutritional. Torix group strains metabolically differ from *R. bellii*-related strains by having fewer lost genes and having additional pathways involved in glycolysis, glucose metabolism, pentose phosphate pathways, and methionine salvage pathway. It has been postulated that these additional pathways help Torix group Rickettsia to purloin energy from its host by importing ATP of the host (Wang et al. 2020). Metabolic models involving all combinations of whitefly endosymbionts predict the highest number of complementary metabolites between Hamiltonella and Rickettsia, a combination that is frequently found in nature (Opatovsky et al. 2018; Pan et al. 2012). In contrast to Hamiltonella and Portiera, it also possesses several virulence factors, such as: genes for Lipid A synthesis, involved in bacterial growth and virulence, genes belonging to “surface cell antigen” group, a typical type IV secretion system, tlyC gene, whose product helps evade phagocytosis, and toxin-antitoxin systems (Zhu et al. 2016).

Despite the unclear advantages from its metabolic pathways, clear advantages of Rickettsia infections have been observed in several ways. Rickettsia provides fitness benefits to its host through higher fecundity, higher survival rates, faster development, female-biased sex ratio, and even increased weight of adult whiteflies, although the underlying mechanism is unknown (Cass et al. 2016; Hong et al. 2016; Asiimwe et al. 2014; Himler et al. 2011). Further, Rickettsia infected whiteflies were reported to be more resistant to heat shocks (40 °C). The mechanism seems to be upregulation of genes related to thermotolerance during normal temperatures, which protects whiteflies during periods of high temperature shocks (Brumin et al. 2011). Finally, early evidence exists that Rickettsia is able to protect its host against pathogens by lowering mortality from *Pseudomonas syringae* Van Hall infection (Hendry et al. 2014). The outcome of Rickettsia infection in whiteflies is nevertheless variable, with cases where infection resulted in few changes in whitefly fitness (Bockoven et al. 2020; Chiel et al. 2009a). Primarily positive effects of Rickettsia are also supported by the fact that no studies to date reported clear negative fitness effects of Rickettsia infection. Ghosh et al. (2018) reported reduced fitness in cassava-colonizing *B. tabaci* which were doubly infected with Rickettsia and Arsenophonus. Taken together, Rickettsia seems beneficial to its host. At least in natural systems. The Rickettsia infection status positively correlates with higher susceptibility to insecticides with different modes of action including thiamethoxam, acetamiprid, spiromesifen, and pyriproxyfen insecticides (Pan et al. 2013; Ghanim and Kontsedalov 2009; Kontsedalov et al. 2008). More research is needed to understand the nature of this effect, which may prove useful in boosting the effectiveness of chemical insecticides in whiteflies.

Similarly to Hamiltonella, Rickettsia can also enhance whiteflies’ ability to transmit viruses. Rickettsia-infected whiteflies acquire more virions and retain the virus longer, which resulted in near doubling of TYLCV transmission (Kliot et al. 2014). Higher concentration of Rickettsia in whitefly midgut results in increased concentration of virions in the filter chamber, which is in contrast to an even distribution of the virus in Rickettsia-free whiteflies (Kliot et al. 2014). Densities of Rickettsia also increase when Rickettsia harboring whiteflies feed on TYLCV-infected plants, while densities of Portiera and Hamiltonella are unaffected, indicating a positive feedback loop between TYLCV and Rickettsia (Su et al. 2014b; Li et al. 2011). A recent study used discovery proteomics and showed a strong association between TYLCV vectoring ability and the expression of *Rickettsia* proteins in both MEAM1 and MED species, confirming the results about the role of *Rickettsia* in TYLCV transmission (Kliot et al. 2020). Finally, Rickettsia changes the behavior of its host by increasing whitefly attraction to TYLCV-infected plants and seems to help counteract negative fitness effects associated with TYLCV acquisition (Kliot et al. 2019; Czosnek and Ghanim 2012). Besides TYLCV, cotton leaf curl Multan virus (CLCuMuV) was shown to be retained for longer in Rickettsia-infected Asia II 1 whiteflies compared to uninfected lines (Lei et al. 2019). Whitefly-Rickettsia-TYLCV interactions are even more complex than already poorly understood whitefly–Rickettsia interactions. RNAi silencing of a whitefly defensin-like antimicrobial peptide, named Btdef, resulted in lower titer and expression of TYLCV in whiteflies, as well as lower Rickettsia density (Wang et al. 2017). While these early results are not enough to explain how whitefly regulates its endosymbionts and the viruses its vectors, they do indicate that the whitefly immune system should be in the focus of future studies.

Zooming out from molecular interactions to the continental scale, the dynamic nature of whitefly-Rickettsia system is still present. A dramatic spread of Rickettsia through *B. tabaci* MEAM1 population in southwestern USA was observed taking only six years to increase from 1% of infected individuals to near fixation (Himler et al. 2011). Rapid spread was explained by the increased fitness of Rickettsia-infested whiteflies (Himler et al. 2011). A study comparing expanding Rickettsia populations in the USA with less abundant Rickettsia in *B. tabaci* MEAM1 populations in Israel did not find different Rickettsia groups (Cass et al. 2015). After staying at near-fixation levels for five years, the Rickettsia gradually declined to 36% of the population over the next six years, with follow-up fitness studies showing no benefits from Rickettsia anymore (Bockoven et al. 2020). In Israel, however, over a 10-year survey, *Rickettsia* completely disappeared from MEAM1 populations and reached fixation in MED (Brumin et al. 2019). Benefits of Rickettsia infection in whiteflies are apparently context dependent, and its abundance in whitefly populations fluctuates with poorly understood complex interactions of genetic and environmental factors, with an equilibrium that is below fixation (Bockoven et al. 2020; Cass et al. 2015; Asiimwe et al. 2014).

The dynamics of Rickettsia are strongly affected by the genotype of its host. The MEAM1 biotype of *B. tabaci* shows both higher Rickettsia density and higher plant-mediated transmission ability than *B. tabaci* MED (Li et al. 2017b). Crossing experiments between two inbred lines of MEAM1 demonstrated that the host nuclear genotype was a sole factor responsible for differing Rickettsia densities and contrasting benefits from Rickettsia infection, potentially explaining most of the observed differences globally (Hunter et al. 2017; Cass et al. 2016). Temperature effect on the density of this endosymbiont has also been explored and showed no significant changes at 35 and 40 °C (Shan et al. 2014; Su et al. 2014b). Response to cold treatments in two studies somewhat differed, with Su et al. reporting significant reduction and Shan et al. reporting no change, although with a trend toward lower densities at lower temperatures. The first steps in understanding molecular mechanisms that govern the whitefly-Rickettsia system have just been taken. Brumin et al. (2019) reported that vitellogenin protein is an important influencing factor of Rickettsia density as it is expressed more in Rickettsia colonized midgut compared to the rest of the body. Immunocapture assays showed interaction of Rickettsia and vitellogenin, seemingly supporting evidence from other insects that vitellogenin is involved in the antibacterial immune response, in addition to its primary role as an egg yolk precursor (Brumin et al. 2019). Despite that, RNAi silencing of vitellogenin reduced both its expression levels and Rickettsia density, which contrasts the antibacterial theory but does underline its importance in regulating levels of Rickettsia (Brumin et al. 2019). Further transcriptomic and silencing studies are needed to fully understand the molecular interactions taking place between Rickettsia and its whitefly host.

### *Wolbachia* sp.

Bacteria belonging to the genus *Wolbachia* Hertig are α-Proteobacteria commonly infecting arthropods and nematodes and are closely related to Rickettsia (Kanakala and Ghanim 2019). They have a relatively high genetic diversity and wide range of interactions with their host, as well as across trophic levels. In whiteflies, Wolbachia was first reported in 1998 (Zchori-Fein and Brown 2002). In mosquitoes, Wolbachia causes cytoplasmic incompatibility (CI), a trait that was successfully exploited in mass-release of infected males biocontrol strategy, which following field trials is currently being implemented in several areas of the world (Anders et al. 2018). Presence of Wolbachia in whiteflies sparks instant interest in understanding the possibility of similar biocontrol of whiteflies.

Out of 16 groups of Wolbachia, two have been detected as symbionts of whiteflies: group B and O (Kanakala and Ghanim 2019; Bing et al. 2014; Zchori-Fein and Brown 2002). Numerous phylogenetic studies have demonstrated the existence of several clades within these two groups, and overall phylogeny of Wolbachia is not congruent with the phylogeny of its host (Kanakala and Ghanim 2019; Ji et al. 2015; Tsagkarakou et al. 2012; Ahmed et al. 2009; Zchori-Fein and Brown 2002). Studies of the patterns of global endosymbiont detections and associations with different whitefly species revealed that during whitefly-Wolbachia co-evolution, there have been several Wolbachia acquisitions and horizontal transfers (Kanakala and Ghanim 2019; Nirgianaki et al. 2003). Phylogenetic studies detected that Wolbachia in whiteflies is unusually similar to strains detected in other insects that feed on the leaf surface and in whitefly parasitoids, postulating that horizontal transmission is possible and has occurred even in the past 20 years (Chiel et al. 2014; Ahmed et al. 2013, 2010a; Sintupachee et al. 2006; Zchori-Fein and Brown 2002). Indeed, follow-up laboratory experiments demonstrated horizontal plant-mediated Wolbachia transmission with *in planta* persistence of 50 days, and transmission by non-lethal interaction with parasitoid *Eretmocerus* sp. nr *furuhashii* for 48 h after its contact with Wolbachia-infected whiteflies (Li et al. 2017a; Ahmed et al. 2015). Within whitefly populations, Wolbachia is primarily maternally inherited, and horizontal transmission, while certainly possible, is a secondary method (Kanakala and Ghanim 2019; Shi et al. 2016; Blawid et al. 2015). Presence of multiple groups and evidence of recent horizontal transfers hint at the possibility of whiteflies to be infected with Wolbachia strains that are not naturally found in whiteflies, potentially including pathogenic strains (Kanakala and Ghanim 2019).

Another similarity with Rickettsia is its localization characterized by a variable phenotype. Wolbachia is detected either confined to bacteriocytes or simultaneously present in bacteriocytes and scattered thought the hemolymph and other organs (Shi et al. 2016; Blawid et al. 2015; Bing et al. 2014; Skaljac et al. 2010; Gottlieb et al. 2008; Li et al. 2007). As with Rickettsia, the scattered phenotype is likely an important enabler of horizontal transmission (Bing et al. 2014). Plant mediated horizontal transmission would be a desirable trait for a potential biocontrol Wolbachia strain, as it could increase transmission in whitefly populations. Co-infection with two different Wolbachia strains in the single individual has also been reported, albeit being rare (Li et al. 2017a). Population studies have shown that Wolbachia-Hamiltonella co-infection is common, but Wolbachia-Rickettsia co-infection is rare (Zchori-Fein et al. 2014).

The genome of Wolbachia is 1.25–1.31 mega base pairs long coding around 979–1339 genes (Genome [Internet] 2020; Opatovsky et al. 2018). Its genome has been less extensively explored than that of previously discussed endosymbionts. Genomic modeling based on Wolbachia’s genome reveals that Wolbachia is a NAD + dependent bacterium, much like Rickettsia (Opatovsky et al. 2018). Competition for NAD + might explain rare co-infection with these two endosymbionts (Opatovsky et al. 2018; Zchori-Fein et al. 2014). While amino acid biosynthesis pathways are absent, Wolbachia is able to produce riboflavin, flavin adenine dinucleotide, and folate which might be of nutritional value to its host (Santos-Garcia et al. 2018). It is possible that Wolbachia could also be involved in synthesis of asparagine, glycine, and precursors of methionine and purine/thiamine (Opatovsky et al. 2018). The genome of Wolbachia from *Aleurodicus dispersus* additionally contains two incomplete prophage sequences (Santos-Garcia et al. 2018).

Understanding which role Wolbachia plays as endosymbiont of whiteflies is currently limited by very few studies demonstrating its biological effects. A single study compared Wolbachia-infected whiteflies with Wolbachia-free populations obtained by means of antibiotic treatment. Results suggest fitness benefits from Wolbachia caused by faster development, increased survival rates, and improved adult longevity (Xue et al. 2012). Further, the same authors report lower performance of parasitoids, probably resulting from decreased body size of 4^th^ instar nymph, which is in agreement with the finding of Ahmed et al. (Ahmed et al. 2015; Xue et al. 2012). Based on these results, Wolbachia isolated from whiteflies definitively does not cause cytoplasmic incompatibility, and on the contrary, it likely provides fitness benefits. This disproves the early speculations that Wolbachia might be driving whitefly speciation and threatens to shatter all hopes of using it as biocontrol agent (Nirgianaki et al. 2003; De Barro and Hart 2000). Nevertheless, all hopes of using Wolbachia-induced CI as a control method in whiteflies are not lost yet. Indeed, a Wolbachia strain isolated from the parasitic wasp *Scleroderma guani* Xiao et Wu is able to cause CI in *B. tabaci* when artificially injected and is transmitted to the offspring (Zhong and Li 2014). The potential use of this strain has been proposed as a control agent in mass release of infected males strategy (Zhong and Li 2014).

### *Arsenophonus* sp.

Arsenophonus stands out from other endosymbionts by its ability to produce B vitamins and decrease virus transmission of its host. It belongs to γ-Proteobacteria, and its first detection in whiteflies was in *Aleurodicus dugesii* Cockerell in 2001, shortly followed by detection in other whitefly species (Gómez-Díaz et al. 2019; Kapantaidaki et al. 2015; Zchori-Fein and Brown 2002). Worldwide phylogenetic studies reveal high genetic diversity of this endosymbiont, especially in the *B. tabaci* complex*,* consisting of two distinct genetic groups named A1 and A2, with the latter being split in multiple sub-clades (Kanakala and Ghanim 2019; Tang et al. 2018; Gueguen et al. 2010). The Arsenophonus phylogeny also reveals evidence of multiple acquisition events and possible history of horizontal transfers (Ahmed et al. 2013; Mouton et al. 2012; Thao and Baumann 2004b). A potential, more distantly related Arsenophonus group has been detected in the ash whitefly (*Siphoninus phillyreae*) (Skaljac et al. 2017).

Although it is regularly detected in endosymbiont surveys, little is known about its biology. Arsenophonus seems to usually inhabit bacteriocytes, in the same manner as Hamiltonella with only one reported exception (Marubayashi et al. 2014; Skaljac et al. 2013; Gottlieb et al. 2008). Namely, Rana et al. (2012) reported Arsenophonus detection in salivary glands and the midgut of *B. tabaci* Asia II species. A limited number of studies point towards lower fitness in Arsenophonus-infected whiteflies through lower adult emergence, increased longevity, and lower fecundity (Ghosh et al. 2018; Raina et al. 2015). Those two studies report conflicting evidence on Arsenophonus effect on whitefly development time. Arsenophonus is also speculated to interact with certain nuclear alleles of its host, and potentially also with Cardinium to result in manipulation of reproductive compatibility between two species of the *B. tabaci* complex (Thierry et al. 2011). Bacteriocyte-confined phenotype and apparent negative fitness effects on its host seem counterintuitive. More research is needed to understand if Arsenophonus indeed manages to enjoy the comfort of bacteriocytes without benefiting its host.

In cassava whiteflies, besides fitness costs, Arsenophonus also decreases acquisition and retention of East African cassava mosaic virus-Uganda (EACMV-UG) (Ghosh et al. 2018). Additionally, cotton leaf curl virus (CLCuV) particles were shown to interact with Arsenophonus GroEL protein in the same study that reported Arsenophonus localization in salivary glands. Potential involvement in transmission of this virus certainly exists but has not been practically demonstrated to date (Rana et al. 2012). Understanding the mechanism of Arsenophonus-virus interactions could be exploited in creating more resistant cultivars, perhaps by *in planta* expression of key proteins of Arsenophonus (such as GroEL).

In other insects, Arsenophonus species were shown to decrease insecticide resistance in *Nilaparvata lugens* Stål, cause mortality of males in *Nasonia vitripennis* Walker, and provide nutritional benefits to their aphid host (Tian et al. 2019; Pang et al. 2018; Taylor et al. 2011). As with Rickettsia and Wolbachia, these observations are yet another example of how closely related bacterial symbionts that are often functionally distinct from each other have very different interactions with their hosts.

A high-quality genome of Arsenophonus from whiteflies of *B. tabaci* species complex is not yet available. Nevertheless, three Arsenophonus genomes from *Aleurodicus dispersus*, *Aleurodicus floccissimus*, and *Trialeurodes vaporariorum* are available and have been used in a comparative genomics study (Santos-Garcia et al. 2018). Strikingly, the genome size of *A. dispersus* strain was only 670 kilo base pairs and around 400 genes, in sharp contrast with 3 mega base pairs in strains from *A. floccissimus* and *T. vaporariorum,* whose genomes code 1880 and 2312 genes, respectively (Santos-Garcia et al. 2018). Consequentially, the highly reduced genome of *A. dispersus* strain has most of its metabolic pathways shared with its host, resembling a typical primary endosymbiont (Santos-Garcia et al. 2018). It is very likely that the two lineages of Arsenophonus differently affect their hosts. More detailed characterization of Arsenophonus symbionts coupled with Arsenophonus elimination studies are needed to fully understand its roles and costs for its hosts. The presence of synthetic pathways for B vitamins sets Arsenophonus apart from other endosymbionts. Both Arsenophonus lineages have pathways for producing vitamins B6 and B2, while *A. dispersus* strain is additionally able to synthetize precursors of vitamins B1 and B9 (Santos-Garcia et al. 2018). It is worth mentioning that the biosynthetic pathways of flavin mononucleotide and flavin adenine, and potentially also of folate are preserved in the other two strains (Santos-Garcia et al. 2018). Taken together, production of vitamins and cofactors, especially vitamin B, seems critical in Arsenophonus and might present the “missing” benefit to its host.

### *Candidatus* Cardinium hertigii

*Candidatus* Cardinium hertigii, previously known as cytophaga-like organism (CLO), belongs to the class Cytophagia. It is estimated to infect cells of 6–7% of all arthropods and also infects certain nematodes (Zchori-Fein et al. 2004). Cardinium detected in whiteflies is most closely related to Cardinium detected in whitefly parasitoid wasps, which immediately raises questions about its involvement in multitrophic interactions (Weeks et al. 2003). Four genetic groups of Cardinium were detected in whiteflies and show incongruence with their whitefly host (Kanakala and Ghanim 2019).

Localization of this endosymbiont using FISH and TEM techniques showed distribution in bacteriocytes and scattered through the body simultaneously (Marubayashi et al. 2014; Skaljac et al. 2010; Gottlieb et al. 2008; Bao et al. 1996; Costa et al. 1995). No studies have been performed to test plant mediated transmission of Cardinium in whiteflies, but the potential exists, as Cardinium was detected in the plant phloem following the feeding of infected *Scaphoideus titanus* Ball (Gonella et al. 2015).

Understanding of its effects on whitefly fitness is very limited. Fang et al. (2014) report lower fitness and lower competitive ability in Cardinium-infected whiteflies. Fitness was reduced by the means of slower development and higher mortality (Fang et al. 2014). Another study compared fitness of Hamiltonella-infected whiteflies with doubly infected whiteflies harboring both Hamiltonella and Cardinium revealing lower fitness in Cardinium-infected populations as explained by lower fecundity, egg hatchability, and number of female offspring (Zhao et al. 2018). Competitive relationships between the two secondary endosymbionts are also suggested by Hamiltonella’s observed gradual displacement from the whitefly population by Cardinium (Zhao et al. 2018).

The genome of Cardinium from *B. tabaci* MED is composed of a 1.01 Mb long chromosome and 52 kb long circular plasmid, coding for 709 and 30 genes, respectively (Santos-Garcia et al. 2014a). Cardinium does not have glycolytic pathways and therefore relies on its host for ATP production (Opatovsky et al. 2018). Rickettsia is also ATP dependent and is very rarely detected in co-infection with Cardinium (Park et al. 2012; Singh et al. 2012). Cardinium’s genome is also characterized by the loss of a biotin biosynthetic pathway and pseudogenization of several genes as a specific adaptation to an endosymbiotic lifestyle (Santos-Garcia et al. 2014a). Pairwise modeling of metabolite complementation between whitefly endosymbionts predicted a lower number of complementarities for Cardinium, possibly suggesting competitive interaction with other secondary endosymbionts, which would support the observed competitive relationship with Hamiltonella (Opatovsky et al. 2018). Comparison with closely related non-whitefly-borne Cardinium strains reveals the curious presence of four genes associated with gliding mobility, four duplications of rtxBDE, and presence of the tolC gene related to the type I secretion system. Further, the rtxBDE gene was likely acquired horizontally, and is similar to the RTX toxin transport system of Vibrio. It further has two putative toxin-related genes (CHV_p018 and CHV_p021) of unknown function, with characteristics of bacterial insecticidal proteins and toxins (Santos-Garcia et al. 2014a). Further studies are needed to shed light on their functions and how they fit into the bigger picture of Cardinium-whitefly interactions.

Study of Li et al. made the first step towards exploring how insights from the Cardinium genome translate into changes on the whitefly proteome level (Li et al. 2018a). Cardinium affects levels of proteins related to the development, immune response, and energy metabolism of *B. tabaci*. Specifically, upregulation of proteins involved in the immune response may indicate the whitefly’s attempt to eliminate or at least control the population of potentially harmful Cardinium. The study of differentially expressed small RNAs resulting from Cardinium infection additionally showed changes in processes related to development, cell apoptosis, and reproduction (Li et al. 2018b). Overexpression of thermotolerance and insecticide resistance-related sRNAs also suggest negative effects of Cardinium infection (Li et al. 2018b). Taken together, available insights show us that Cardinium is a competitive intruder of the whitefly body, still retains virulence factors, and is likely capable of plant-mediated horizontal transmission. This makes Cardinium a good candidate for use as a biocontrol agent, but only if its negative effects on whitefly fitness could be improved, potentially though the means of genome alterations.

### *Candidatus* Hemipteriphilus asiaticus

*Candidatus* Hemipteriphilus asiaticus is one of the least known endosymbionts of whiteflies. It was first detected using the Rickettsia-specific primers but subsequent sequencing and phylogenetic analysis grouped it closer to the genus *Orientia* Tamura (Bing et al. 2013a). It belongs to the α-Proteobacteria class, family Rickettsiaceae. The name *Candidatus* Hemipteriphilus was proposed to reflect the fact that it is found in Hemipteran insects in Asia (Bing et al. 2013b). So far, it has been detected in *B. tabaci* China 1 species from China, Asia I from India, and Asia II-1 and Asia II-7 species from India and Pakistan (Paredes-Montero et al. 2020; Kanakala and Ghanim 2019; Ansari et al. 2017; Bing et al. 2013b). It exhibits a bacteriocyte-confined localization in the whitefly body and is maternally inherited through transferred bacteriocyte (Bing et al. 2013b). Besides its presence and localization, almost nothing else is known. Its genome has not been sequenced, and no comparative studies have been performed. The endosymbiont survey of Paredes-Montero et al. (2020) revealed higher Hemipteriphilus infection rates in urban areas with less pesticide use, but whether this correlation reflects any biological consequence of Hemipteriphilus infection remains to be seen. Understanding of Hemipteriphilus is limited due to its relatively recent detection, which is in turn the result of its low abundance in whitefly populations. In the context of utilizing endosymbionts in whitefly biocontrol, such low abundance endosymbionts could be of interest if their low abundance is a result of reduced fitness of its host. If those endosymbionts could be spread through the population more efficiently, they could act as whitefly antagonists.

### *Candidatus* Fritschea bemisiae

Knowledge about Fritschea is extremely limited, much like in the case of Hemipteriphilus. In 2003, bacteria from the class of Chlamydiae, family Simkaniaceae, were found to infect *B. tabaci* and *Eriococcus spurius,* and upon characterization, two novel species were proposed: *Candidatus* Fritschea bemisiae and *Candidatus* Fritschea eriococcid, respectively (Thao et al. 2003). The sequencing of a 16.6 kb fragment around rRNA coding region allowed for the phylogenetic placement of these bacteria (Thao et al. 2003). The two novel species differ not only in nucleotide identity percentage, but also by a 23S rRNA gene intron which is present only in *Ca*. F. bemisiae (Everett et al. 2005). Their closest known relative is *Simkania negevensis* Everett et al., a potential emerging human pathogen associated with bronchiolitis and pneumonia (Vouga et al. 2017; Thao et al. 2003). Similar to other Chlamydiae, Fritschea has a biphasic lifecycle, forming rod-like reticulate bodies and smaller electron-dense elementary bodies (Everett et al. 2005). Fritschea infects whitefly bacteriocytes, but it is not known what the consequences of this infection are (Marubayashi et al. 2014). It has been detected so far in *B. tabaci* MED, MEAM1, and New World 2 with detections in the United States (CA), Brazil, and Pakistan (Kanakala and Ghanim 2019; Abbas et al. 2017; Marubayashi et al. 2014). If demonstrated to exhibit parasitic characteristics in whiteflies, it might be a potential biocontrol agent, although its similarity with human pathogens will likely result in a higher safety scrutiny.

## Whiteflies endosymbionts and IPM: the terms of a challenge

The most obvious and the most critical target for endosymbiont-based control methods is the whitefly’s obligatory endosymbiosis with Portiera, which if successfully targeted, could cut the supply of critical nutrients that whiteflies need. But Portiera’s tight metabolic integration with its host, makes it a hard target to hit with current antibacterial methods. Portiera evolved far from its ancestor, which led to losses of many metabolic pathways which severely reduces the attack surface. For example, there is a significant effort being put in developing antibiotics that target specific enzymes of bacterial central metabolism, but Portiera lacks components of citrate cycle and glycolysis pathway (Horowitz et al. 2020; Opatovsky et al. 2018; Murima et al. 2014). While it still may be possible to disrupt its role, such efforts likely necessitate methods which go beyond conventional antibiotics with broad effect.

Effective disruption of Portiera necessitates an approach that is more similar to targeting a metabolic process of its whitefly host than trying to “cure” a bacterial infection using antibiotics. This is due to its heavily reduced metabolism, which is shared with the host and has fewer metabolic targets compared to more complex bacteria. A reduced metabolism of Portiera also means fewer metabolic redundancies, which might favor such a specific method. In more complex bacteria, such as human pathogen Salmonella, redundancies in many metabolic pathways limit the development of new antimicrobial compounds (Becker et al. 2006). Besides, effective delivery of any molecule is difficult as Portiera enjoys special protection that bacteriocytes provide. Emerging technologies such as RNA interference (RNAi) may be an effective tool to specifically target critical components of whitefly-Portiera interactions. Tissue-specific RNAi for gene silencing in *B. tabaci*, and effective use of RNAi as a method to silence several genes has already been demonstrated (Ghanim et al. 2007). The effective delivery of RNAi is likely going to be a significant roadblock to widespread use. One potential solution could take advantage of whitefly feeding behavior and deliver dsRNA through the plant phloem, as has been previously demonstrated (Kanakala and Ghanim 2016). Alternatively, engineered secondary endosymbionts could be used to deliver dsRNA directly to bacteriocytes; however, this approach requires developing methods for in vitro culturing the engineered endosymbionts, a goal that has not been yet reached. At the moment, RNAi in whiteflies is still under development and is limited mostly by difficult delivery and easy degradation of dsRNA in the environment and the insect body (Jain et al. 2021; Christiaens and Smagghe 2014). Still, incremental improvements in the field continue to being made. For example, use of carbon quantum dots conjugated to dsRNA and additional knockdown of gut nucleases that are responsible for degradation of dsRNA have recently been explored (Kaur et al. 2020). Looking ahead, a COVID-19-fueled explosion of RNA research, especially on the topic of RNA delivery, will undoubtfully have a profound impact on RNA use in the fields outside human medicine (Coccia 2021; Cheng et al. 2020; Miao et al. 2019; Pardi et al. 2018).

Secondary endosymbionts have potential uses beyond serving as delivery vectors for molecules. Endosymbiotic bacteria with pathogenic or other characteristics that can control whiteflies could have a potential advantage over classic biocontrol pathogens as they can be more persistent due to their maternal inheritance and horizontal transmission channels. Endosymbionts that would prevent virus transmission could have even greater advantage over classic biocontrol agents as they could persist in the population much longer than pathogenic endosymbionts. The most significant success story in exploitation of insect endosymbionts is certainly the use of Wolbachia-infected mosquitoes for successful control of Dengue fever on a large scale (Anders et al. 2018; Hoffmann et al. 2011). Similar success in whiteflies still seems as a distant goal, but the potential certainly exists. An example of *Scleroderma guani* sourced Wolbachia which persists in whitefly populations and causes cytoplasmic incompatibility is a prime example (Zhong and Li 2014). Hamiltonella provides fitness benefits to whiteflies and is able to enhance virus transmission properties of its host and to mitigate negative effects that arise from TYLCV infection (Kliot and Ghanim 2013; Su et al. 2013a). This makes Hamiltonella-harboring populations even more economically devastating than non-Hamiltonella-harboring populations. Being beneficial to whiteflies makes any efforts to disrupt Hamiltonella certainly welcome. However, being a facultative endosymbiont renders its elimination insufficient to adequately resolve problems that arise from whitefly infestation. Instead, by studying how Hamiltonella provides benefits, we can learn more about factors and processes that influence whitefly biology and virus transmission, with an aim to target those processes more efficiently. Such work has already proven promising. Understanding of Hamiltonella-TYLCV interactions resulted in the creation of virus resistant plants that express the GroEL protein of this whitefly endosymbiont. Studies of Arsenophonus show how endosymbionts can reduce virus acquisition and retention (Ghosh et al. 2018). With sufficient understanding of these interactions, it might be possible to enhance the virus suppressing abilities of endosymbionts, which could tackle the more economically significant problem: damage from whitefly-borne viruses.

Efforts to understand fitness benefits revealed that Hamiltonella is involved in the production of a not yet identified molecule that suppresses jasmonic acid defense in tomato (Su et al. 2015). Future identification and characterization of this compound will undoubtedly result in better understanding the plant defense regulation. Rickettsia, with its dynamic and highly context-dependent interaction with its host, reminds us that outcomes of harboring a certain endosymbiont are not binary and offers even more opportunities to explore the factors that alter the biology of whiteflies. RNAi silencing was already used to probe the interaction of whitefly vitellogenin protein with Rickettsia, which showed that change in the regulation of endosymbiont levels is possible via silencing in the insect genome (Brumin et al. 2019). Endosymbionts such as Arsenophonus and Cardinium show us that it is possible to invade whiteflies, evolve and establish endosymbiosis while still retaining virulence related genes and causing negative fitness effects (Santos-Garcia et al. 2014a). This leaves us with the following questions: Do we see the full picture? Can we recruit these endosymbionts in our battle to control whiteflies in our agroecosystems? There may exist yet to be observed conditions or ecological niches in which these seemingly parasitic endosymbionts provide benefits to whiteflies, and we certainly have a lot to learn from them. The rarely detected and poorly characterized whitefly symbionts Hemipteriphilus and Fritschea might seem irrelevant in comparison with other globally distributed endosymbionts, but they do spark curiosity when evaluated in the context of our ongoing quest for novel whitefly control methods. Their rarity could be the first evidence of negative impacts on whitefly fitness and competitiveness.

Finally, the sheer number of discovered endosymbionts raise the question of whether the list of seven secondary endosymbiont species is final or not. All large-scale surveys of whitefly endosymbionts are performed using primer pairs that specifically target known endosymbiont species, which limits the discovery of new, especially more phylogenetically distant bacteria. Combined with an expected lower abundance of endosymbiotic bacteria that negatively impact whitefly fitness, the possibility of the future discovery of novel endosymbionts certainly exists.

## From parasites to mutualists: evolution at its best

The rapidly increasing understanding of secondary endosymbionts is shifting our focus from whiteflies as singular entities with defined characteristics to a more holistic view of whiteflies and their populations as holobionts. This system is able to harvest benefits from a diversity of bacteria, while still being able to eliminate them as soon as the context changes and benefits no longer exist. The relationship between whiteflies and endosymbiotic bacteria likely started as a parasitic one and evolved into a regulated, mostly mutualistic one. Evidence of its parasitic origin remain, as witnessed by the examples of deleterious effects of harboring certain endosymbionts under specific conditions. Bacterial infection that proves to be beneficial persists over host generations. Life in a very stable environment leads to co-evolution in which bacteria lose their metabolic plasticity and virulence factors. Finally, adaptations are so specific that bacteria are able to survive not only in one host species, but only in one cell type of its host, appropriately named bacteriocytes.

In whiteflies, we see bacteria at every step of this figurative evolutionary ladder that leads to obligate symbiosis, and perhaps to organellogenesis. Portiera is at the very top of the ladder short of becoming a symbionelle. Regardless of the formal terminology, it is functionally and evolutionarily an essential element of a whitefly. It encodes genes that are not only beneficial, but essential in enabling whiteflies to feed exclusively on the phloem sap (Calle-Espinosa et al. 2016; Santos-Garcia et al. 2012; Sloan and Moran 2012). Hamiltonella is just below, with its bacteriocyte-confined lifestyle, absence of virulence factors, long co-evolution with no detectable horizontal transfers, and perfect phylogenetic congruency with its host. Hamiltonella reaches a step farther than its host and manipulates the defense mechanisms of the plant that its host feeds on, ensuring mutual benefits and its spread (Su et al. 2015). Rickettsia did not yet reach the stage of settling in one host as evidenced by the history of horizontal transfers, multiple genetic groups, and ability to infect even whitefly parasitoids. Presence of virulence factors, toxin-related genes, and a type IV secretion system are also indicative of its lifestyle (Zhu et al. 2016). Even though it is less specifically adapted to one host, Rickettsia is able to provide enough fitness benefits to a whitefly population to enable its invasion on a continental scale (Cass et al. 2016; Hong et al. 2016; Asiimwe et al. 2014; Himler et al. 2011). Not being limited to one host and one cell type is beneficial for Rickettsia as it is able to spread and be present in more diverse environments. It is capable of plant-mediated horizontal transmission and transmission between trophic levels as it is infectious to *Eretmocerus* sp. parasitic wasps. Wolbachia appears to be very similar to Rickettsia on the endosymbiont evolutionary ladder, although further research is needed to fully understand the lifestyle of Wolbachia. Cardinium, while also poorly understood, seems to live a more parasitic life, with reported negative fitness effects on its host. Finally, Hemipteriphilus and Fritschea still have an unknown placement on this figurative evolutionary ladder, although its bacteriocyte confinement (if confirmed to be the only phenotype) suggests either a very close mutualistic, or a highly specialized parasitic relationship with its host.

## Future research

It took several decades of research and technological advances to precisely understand the roles of Portiera. Now, the research on Portiera-whitefly interactions is shifting from understanding the functions to understanding the mechanisms involved in metabolic complementation and the roles of facultative endosymbionts. The fact that whitefly bacteriocytes have a distinct, likely polyploid genome is recent information which will certainly shape future genomic studies in whiteflies. The understanding of protein and metabolite transport systems between endosymbionts and their host is in the early stages. One of the next steps in utilizing this information would be producing separate high-quality genome assemblies for whiteflies and their bacteriocytes, which holds the potential to uncover the adaptations and mechanisms that unite two organisms into one metabolism.

Research on whitefly endosymbionts will continue to accelerate as a more diverse set of methods is available. Use of highly specific and sensitive chromatography and spectrometry methods to analyze metabolites in comparative studies involving whiteflies with different endosymbiont compositions will aid genomic modeling studies. Fitness and transcriptome studies under controlled differing environmental conditions will help us go beyond the conclusions of “context-dependent” associated with secondary endosymbionts. Proteomic studies have just begun to be applied to this system and present yet another much needed approach. Culture of whitefly endosymbionts, with the help of insect cell culture, will enable an even closer look at the molecular interactions and allow detailed metabolic studies. First cell cultures of Arsenophonus endosymbiont from the pigeon louse fly *Pseudolynchia canariensis* (Macquart) have already been established (Dale et al. 2006). Such cultures outside the insect body allow easier genetic transformation of these bacteria which is critical to characterizing endosymbiont-specific genes and potentially creating improved biocontrol agents. Use of locked nucleic acid probes (LNA) in localization of endosymbionts with further optimization of the technique can allow single-cell sensitivity and a more detailed understanding of phenotypic variation in secondary endosymbionts (Priya et al. 2012).

A significant hurdle in understanding the effects of secondary endosymbionts on the macroscopic scale is the lack of methods for their stable and selective elimination. A major breakthrough on this topic has recently been made by delivering antibiotics through the plant, but the method is still not selective for all secondary endosymbionts (Zhao et al. 2019). A novel or highly improved technique is needed. RNA interference targeting specific endosymbiont transcription holds the potential to achieve highly needed selectivity and efficacy (Grover et al. 2019). Such a method combined with the already successfully used microinjection and/or plant-mediated silencing technique would allow interaction studies between whiteflies with any genetic composition and any endosymbiotic bacteria (Zhong and Li 2014). The recent development of CRISPR/Cas9-based genome editing in *B. tabaci* allows even more precise studies (Heu et al. 2020).

## Conclusion

Damages that arise from whitefly infestations continue to be a major problem in systems ranging from subsistence farming in the open field to highly intensive greenhouse production. As climate change continues to modify our environment, different species are favored by these new conditions, leading to hard-to-predict changes in both natural and manmade ecosystems. Nevertheless, species that can adapt quickly and that colonize a wide range of environments have a competitive advantage in this rapidly changing environment. It is safe to say that the wide range of endosymbionts provides adaptive plasticity to whiteflies. Whitefly species take advantage of newly favorable environments and fast global transport of plant material, and with additional help from their unique bacterial toolkit of superpowers, they are able to mount devastating invasions of our agricultural systems. Challenging control prompts the need for novel methods and sets the research community on an exploratory journey which includes whitefly endosymbionts. Taken together whitefly endosymbionts represent an evolutionary ladder which offers us valuable insights at every step. Endosymbionts such as Portiera, with very tight metabolic integration with its host, are an excellent target for disruption. More recently acquired bacteria on the other hand retain many characteristics from their parasitic past which might just be enough to use to our advantage for exploitation as biocontrol agents. Those in between the two teach us about complex regulatory mechanisms, not only inside the insect’s body, but also extending to the regulation of plant defense pathways. Technological advances in the areas of biocontrol, antibiotics, -omics, genetic manipulation, and gene silencing allow us to learn from and apply the offered lessons. The progress is clear, but many important questions remain: How does the journey from being a parasite to becoming a mutualist look? Can we gain enough knowledge to utilize endosymbionts in whitefly control? Are the most useful endosymbionts for biocontrol yet to be discovered? Which external factors affect and regulate whitefly-endosymbiont interactions? Answering these questions will bring us closer to reaching the ever-elusive goal of developing novel, more targeted, whitefly control strategies. Finally, it will expand our understanding of not only whiteflies, but biological interactions in general.

## Authors' contributions

MM and CR conceptualized the idea for the review. MM performed the literature search and wrote the manuscript draft. MG, LH, and CR critically revised the manuscript and contributed to the text. All authors read and approved the final manuscript.
